# *De novo* assembly and characterization of tissue specific transcriptomes in the emerald notothen, *Trematomus bernacchii*

**DOI:** 10.1186/1471-2164-14-805

**Published:** 2013-11-20

**Authors:** Troy J Huth, Sean P Place

**Affiliations:** 1 Department of Biological Sciences, University of South Carolina, Columbia, SC, USA; 2 Environment and Sustainability Program, University of South Carolina, Columbia, SC, USA

## Abstract

**Background:**

The notothenioids comprise a diverse group of fishes that rapidly radiated after isolation by the Antarctic Circumpolar Current approximately 14–25 million years ago. Given that evolutionary adaptation has led to finely tuned traits with narrow physiological limits in these organisms, this system provides a unique opportunity to examine physiological trade-offs and limits of adaptive responses to environmental perturbation. As such, notothenioids have a rich history with respect to studies attempting to understand the vulnerability of polar ecosystems to the negative impacts associated with global climate change. Unfortunately, despite being a model system for understanding physiological adaptations to extreme environments, we still lack fundamental molecular tools for much of the Nototheniidae family.

**Results:**

Specimens of the emerald notothen, *Trematomus bernacchii,* were acclimated for 28 days in flow-through seawater tanks maintained near ambient seawater temperatures (−1.5°C) or at +4°C. Following acclimation, tissue specific cDNA libraries for liver, gill and brain were created by pooling RNA from n = 5 individuals per temperature treatment. The tissue specific libraries were bar-coded and used for 454 pyrosequencing, which yielded over 700 thousand sequencing reads. A *de novo* assembly and annotation of these reads produced a functional transcriptome library of *T. bernacchii* containing 30,107 unigenes, 13,003 of which possessed significant homology to a known protein product. Digital gene expression analysis of these extremely cold adapted fish reinforced the loss of an inducible heat shock response and allowed the preliminary exploration into other elements of the cellular stress response.

**Conclusions:**

Preliminary exploration of the transcriptome of *T. bernacchii* under elevated temperatures enabled a semi-quantitative comparison to prior studies aimed at characterizing the thermal response of this endemic fish whose size, abundance and distribution has established it as a pivotal species in polar research spanning several decades.

The comparison of these findings to previous studies demonstrates the efficacy of transcriptomics and digital gene expression analysis as tools in future studies of polar organisms and has greatly increased the available genomic resources for the suborder Notothenioidei, particularly in the Trematominae subfamily.

## Background

Perciform fishes of the suborder Notothenioidei comprise a major portion of the Southern Ocean fauna [1,2]. They began to radiate into Antarctic waters in the early Tertiary, gradually adapting to the progressive cooling, which set in after the opening of the Drake passage and the formation of the circumpolar current some 14–25 million years ago [2,3]. Isolation of the Antarctic continental shelf by the Polar Front has produced arguably the coldest, most oceanographically stable environment on the planet. However, in direct opposition to this highly stenothermic environment are the profound environmental extremes produced by the transition from 24 hours of sunlight to complete darkness over the winter months, resulting in significant variation in primary productivity. As a result, Antarctic marine organisms inhabiting these ice-laden waters have faced unique metabolic and physiological challenges for survival and persistence. The impacts of low temperatures and seasonally limited food availability have long been recognized as primary selective forces driving the evolution of the many endemic species found in Antarctica today [4-8]. In addition to the high degree of endemism produced by these evolutionary processes, a wide-array of functional adaptations have been fixed among protein families of several Antarctic fish, including chaperonins [9], heat shock proteins [10,11], heme proteins [12,13], tubulin kinetics [14], and anti-freeze proteins [15,16]. This rigid oceanographic stability however, may have resulted in an ecosystem filled with endemic fauna that are poorly poised to deal with rapid climate variation [7,17]. For instance, cold specialization has resulted in increased mitochondrial densities at uncompensated capacities in some notothenioids [18-20]. These increased densities have also been combined with reductions in hematocrit and cardiovascular output [21,22].

Although a significant amount of sequencing work has been done to elucidate the evolutionary history and phylogenetic relationships among these unique fishes, much of the available sequence information is constrained to a few highly conserved genes such as ribosomal and mitochondrial genes, or highly specified genes such as the antifreeze glycoprotein genes. Recent advances in DNA sequencing technology have lead to a significant increase in the availability of molecular tools to ecologists and physiologists. A particular research niche that is poised to benefit greatly from this rapid increase in sequence data is the field of polar biology. The availability of well-annotated transcriptomes from a variety of polar species will provide the groundwork for future functional genomics studies aimed at elucidating the impact of global climate change on polar ecosystems. With the application of next generation sequencing tools in an ecological setting, we can begin to investigate organismal responses at a level of complexity that was not approachable in years past. To date, only two large-scale sequencing studies of transcribed genes have been published for any Antarctic notothen, including an EST library for *Dissostichus mawsoni* in the subfamily Pleuragramminae [23] and a comparative study of the transcriptomes from a member of the Nototheniinae and Pleuragramminae sub-families [24]. To date, relatively little sequence information is available for any member of the Trematominae subfamily despite the ecological importance of these fish in coastal Antarctic environments. Due to its circumpolar distribution, abundance and relative ease of collection, *Trematomus bernacchii* has long been a target species for physiological and biochemical studies assessing the plasticity of this highly stenothermic family of fishes and the cost of adaptation to such a cold stable environment [10,11,25-30]. While earlier attempts at using heterologous hybridizations to a cDNA array of a temperate goby provided insight into the transcriptional response of highly conserved genes in this species [31], we still lack a robust approach to functional genomics in these unique fish. Here we describe the *de novo* assembly and annotation of the transcriptome of the emerald notothen, *T. bernacchii*. In addition, we provide a glimpse into the tissue specific response to thermal stress at the level of the transcript that highlights the sensitivity and utility of these applications in polar fish.

## Results and discussion

### Sequence data and de novo assembly

**Figure 1:**
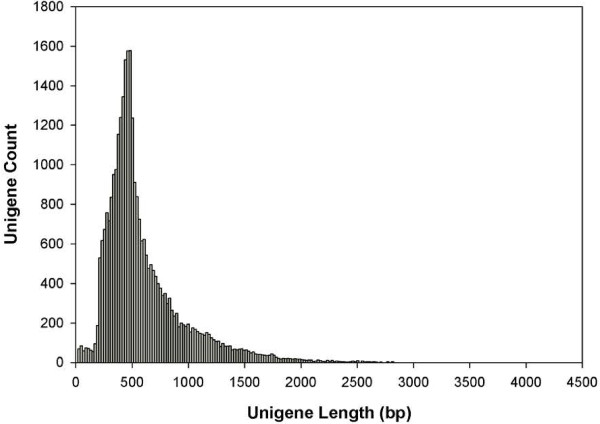
**Unigene length distribution results of *de novo *assembly.** Length distribution of unigene sequences obtained from the *de novo* assembly. The unigenes are grouped from shortest to longest with each column representing the number of unigenes of that specific length.

Roche 454 sequencing generated a total of 738,379 unpaired reads across six samples with an average length of 335.80 bp. The raw data were deposited at the NCBI Sequence Read Archive under the study accession number [SRP026018]. After sequence trimming for adapter removal and screening of sequences based on quality and ambiguity scores, 735,507 reads remained with an average length of 320.98 bp. *De novo* assembly of these reads using the CLC Genomics *de novo* assembly tool matched 468,721 reads, leaving 266,786 unmatched singletons. The final transcriptome contained 30,107 unigenes with an average length of 605 bp (Table 1, Figure 1).

**Table 1:** **Read sequencing and *de novo *** **assembly statistics**

**Sequencing and assembly statistics**	
Reads sequenced	738,379
Reads after screening	735,507
Assembled reads	468,721
Singletons reads	266,786
Unigene count	30,107
Average unigene length (bp)	605
Coverage (X)	6.45
N75	466
N50	671
N25	1,085

Overall, the resulting number of unigenes produced in our assembly is in line with results reported in previous studies utilizing Roche 454 data for *de novo* full transcriptome assemblies of other fish species, including 1,150,339 reads yielding 36,811 unigenes with an average length of 888 bp in common carp (*Cyprinus carpio*) [32]; and 1,004,081 reads yielding 33,191 unigenes with an average length of 991 in Asian seabass (*Lates calcariefer*) [33]. Furthermore, our average coverage (6.45X) is comparable to 454 *de novo* transcriptome assemblies conducted with similar number of sequencing reads [34]; and possesses a similar percentage of reads mapping back to the unigenes (63.7%) as well [34,35].

**Figure 2:**
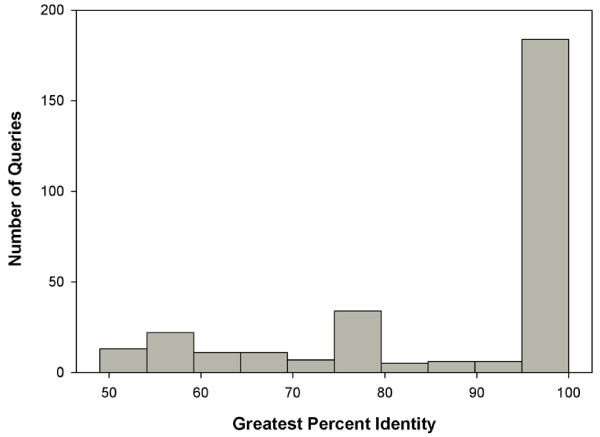
**BLAST results: *T. bernacchiii *protein sequences vs. unigene sequences.** The percent identity of each known *T. bernacchii* full or partial protein sequence versus a unigene from the assembled transcriptome. 299 of 303 query sequences of known *T. bernacchii* protein sequences matched a unigene. 236 of the queries aligned with an *e*-value < 10^-10^ and of those 184 matched a unigene at greater than 95% identity.

To further validate our transcriptome, we compared the alignment and similarity of our unigenes to the 303 known full and partial *T. bernacchii* sequences available on NCBI at the time of our study using BLASTx. This comparison yielded 236 matches with an *e*-value < 10^-10^, of which 180 sequences possessed a percent identity > 95% (Figure 2, Additional file 1: Table S1). Despite the limited sequence data available for *T. bernacchii,* these alignment metrics indicate a relatively accurate transcriptome assembly.

Of the 303 *T. bernacchii* sequences available, only four failed to align to a unigene sequence. Two of these sequences form interphotoreceptor retinoid-binding protein (IRBP), a protein that accumulates in the subretinal space and facilitates exchanges during the “visual cycle” [36]. Due to the high degree of specificity to the visual process, it is unlikely that this product would be found in the liver, gill or brain tissues that were sequenced in this effort. Another unmatched query represented a partial of E3 ubiquitin protein ligase 2; however, six different unigenes (1260, 4958, 15902, 18843, 22152, and 27842) matched E3 ubiquitin ligase-like proteins during annotation. It is likely that this partial query sequence did not overlap with the available assembled sequence of the unigenes, which themselves are not complete representations of the entire coding region of E3 ubiquitin ligases. The last query that failed to align was that of a putative voltage-activated sodium channel alpha subunit (SCNA), which are functionally conserved and have lengths ranging between 3300-3500 bp in teleost fish [37]. As the partial sequence available for *T. bernacchii* SCNA is only 276 bp, it is very likely our sequencing efforts simply failed to provide areas of overlap with the partial cDNA sufficient to align it to a unigene.

In a effort to further validate our transcriptome assembly, we compared our assembly to the transcriptomes of nine other prior sequenced teleost fish including: *Notothenia coriiceps, Chaenocephalus aceratus, Pleuragramma antarcticum, Gasterosteus aculeatus, Takifugu rubripes, Oryzias latipes, Tetraodon nigroviridis, Oreochromis niloticus* and *Danio rerio* (Table 2)*.* BLAST searches were conducted using our assembled unigenes as query sequences against the available transcriptome of each fish using either BLASTx or BLASTn, depending on the form of the sequence database available. The BLASTn results demonstrated a high degree of similarity between the transcriptome generated for *T. bernacchii* and that of *N. corriceps*, a related notothenioid species (19,253 hits *e*-value < 10^-3^, 15,861 hits *e*-value < 10^-10^), as well as strong similarity to the two other more distantly related Antarctic fish, *P. antarticum* (16,822 hits *e*-value < 10^-3^, 10,033 hits *e*-value < 10^-10^) and *C. aceratus* (14,533 hits *e*-value < 10^-3^, 10,138 hits *e*-value < 10^-10^). BLASTx searches against the non-polar fish in our analysis yielded results similar to previous comparisons between the transcriptomes of notothenioid fishes and those of non-polar fish species (Table 2) [38].

**Table 2:** ***T. bernacchii *** **unigene BLAST comparison against 9 teleost fish**

**Species**	**Sequences available for subject species**	***T. bernacchii *** **hit** ***e*-Value < 10**^**-3**^	***T. bernacchii *** **hit** ***e*-Value < 10**^**-10**^
*Notothenia coriiceps*	34,371	19,235	15,861
*Chaenocephalus aceratus*	17,233	14,533	10,138
*Pleuragramma antarcticum*	19,945	16,822	10,033
*Gasterosteus aculeatus*	27,576	9,056	8,010
*Takifugu rubripes*	47,841	8,539	7,541
*Oryzias latipes*	24,674	8,686	7,630
*Tetraodon nigroviridis*	23,118	8,382	7,393
*Oreochromis niloticus*	26,763	9,116	8,066
*Danio rerio*	43,309	8,972	7,800

BLASTx searches were conducted for *Gasterosteus aculeatus, Takifugu rubripes, Oryzias latipes, Tetraodon nigroviridis, Oreochromis niloticus,* and *Danio rerio*; BLASTn searches were conducted for: *Notothenia coriiceps, Chaenocephalus aceratus,* and *Pleuragramma antarcticum*, due to a lack of available protein databases.

### Annotation, classification, and analysis

BLASTx results using the unigene sequences as queries against the non-redundant protein sequences (nr) database at NCBI yielded 9,243 significant alignments (*e*-value 10^-3^), and when combined with an additional 3,760 BLASTn results from our searches against the nucleotide collection (nr/nt) database, yielded a total of 13,003 unigene sequences with a significant BLAST result. Of these results, 7,677 unigene sequences mapped to GO terms using the BLAST2GO (B2GO) software package. Ultimately, InterProScan results merged with B2GO annotations yielded a total of 6,528 fully annotated unigene sequences. The final distribution of annotated unigenes included 5,351 unigenes sequences with a significant BLAST result only, 1,149 unigene sequences mapped in B2GO, and 6,528 unigene sequences fully annotated with GO terms (Additional file 2: Table S2). Overall, approximately 43% of our unigenes had a significant hit in the nr protein or nucleotide database and we were able to assign GO annotation to 21.6% of unigenes in the dataset, which was highly comparable to recent assembly and annotation efforts for other non-model fish species [24,38,39].

### Tissue specific gene expression

**Figure 3:**
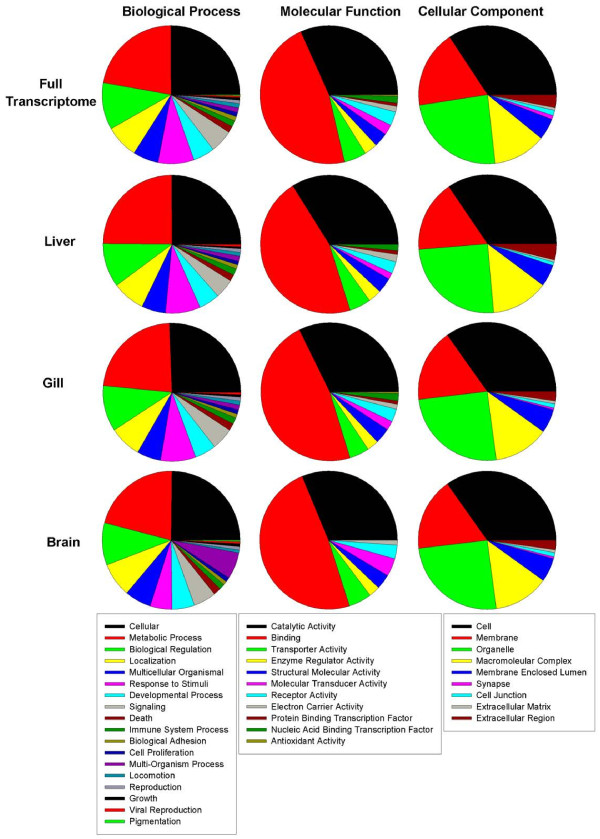
**Tissue specific GO comparison.** Unigenes expressed in at least one treatment were included in the gene ontology analysis. GO terms were determined using BLAST2GO [40] with an *e-*value cut off of 10^-5^, a minimum sequence filter of 25, and sorted based on level 2 GO classifications. Any GO term that met the filter for the minimum number of sequences to include as a node (n = 25) was included in the comparison.

The transcriptome wide gene ontology in WEGO format yielded 14,181 (~35%) GO annotation results for cellular component, 17,515 (~44%) GO annotation results for biological process, and 8,447 (~21%) GO annotation results for molecular function in *T. bernacchii* (Additional file 3: Table S3). For the most part, expression levels associated with individual GO classifications were found to be very similar across the entire transcriptome and individual tissues (Figure 3, Additional file 4: Table S4). However, some gene expression trends can be detected that are likely associated with differences in functional roles of the tissues. For example, when comparing the biological processes between tissues, the role of the liver in protein metabolism is evident with 24.7% of its total biological processes dedicated to metabolic processes as compared to 22.9% in the gill and 21.1% in the brain. Also, the gill tissue demonstrated elevated representation for the GO classification ‘response to stimuli’, perhaps due to their more immediate exposure to the external environment, with approximately 8.2% of biological processes dedicated to this function as opposed to 5.1% in the brain.

The molecular function of expressed transcripts also displayed variation that mirror physiological differences between the tissues. For instance, despite being a primary site for cellular detoxification, liver did not exhibit sufficient antioxidant activity to meet the applied threshold (n ≥ 25 transcripts) for this GO term, whereas the GO term antioxidant activity was highly represented in the gill, perhaps due to this tissue’s direct contact with the extremely cold, highly oxygenated water of the Southern Ocean (Figure 3). These results parallel previous expression patterns of antioxidant proteins in the gill and liver tissue of *T. bernacchii*; Enzor and Place [40] report protein concentrations of two isoforms of the antioxidant enzyme family superoxide dismutase (SOD1 and SOD2) are relatively higher in gill tissue compared to liver tissue and total SOD activity is also elevated in gill tissue. As for cellular components, each tissue largely mirrored one another and the transcriptome as a whole (Figure 3). Enzyme code distributions for each tissue calculated by the main enzyme classes: oxidoreductases, transferases, hydrolases, lysases, isomerases, demonstrated a similar pattern of enzyme activity across tissues. The distribution being: liver (359, 542, 559, 74, 71, 127), gill (252, 269, 308, 39, 44, 72) and brain (245, 355, 377, 52, 59, 93) respectively (Additional file 5: Figure S1). Notably, the liver displayed the greatest variety of enzymes, commiserate with its involvement in protein and lipid metabolism.

**Figure 4:**
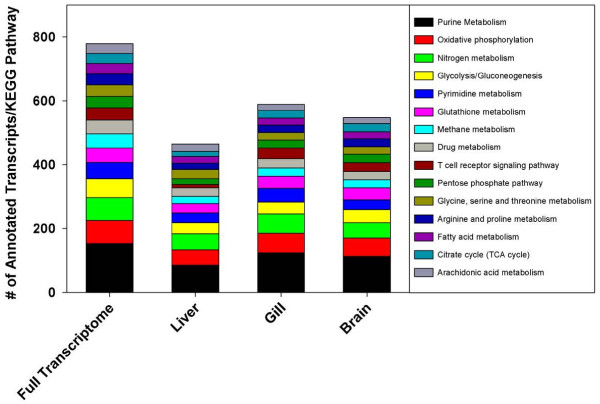
**Categorization of *T. bernacchii *unigenes to KEGG biochemical pathways.** A KEGG biochemical pathway analysis was performed on the full transcriptome and individual tissue transcriptomes using any unigene expressed in at least one treatment. The stacked columns represent the top 15 KEGG pathways found in the full transcriptome and their distribution in each tissue, using an *e-*value cut off of 10^-5^ and minimum pathway assignment cutoff of 30.

A comparison of the unigenes against the KEGG database, a resource for understanding high-level functions and utilities of biological systems [41], yielded 1,948 sequences with a significant match (BLASTx *e*-value < 10^-5^) to 119 KEGG pathways and 784 enzymes. Of the 119 pathways there were 62 pathways with over 10 sequences assigned. The pathway for purine metabolism was the most commonly assigned (153, 7.85%), followed by oxidative phosphorylation (73, 3.75%), nitrogen metabolism (71, 3.64%), glycolysis/gluconeogenesis (59, 3.03%), pyrimidine metabolism (51, 2.62%), glutathione metabolism (45, 2.31%), methane metabolism (45, 2.31%), drug metabolism- other enzymes (43, 2.2%), T-cell receptor signaling pathway (38, 1.95%), and pentose phosphate pathway (36, 1.85%), with the remaining 119 pathways listed in the supplemental information (Additional file 6: Table S5). When analyzing dominant KEGG pathways assigned to the unigenes expressed in each tissue, the most commonly assigned KEGG pathways generally followed the most commonly assigned pathways for *T. bernacchii* as a whole, with a few exceptions (Figure 4). Overall, liver tissue matched 1,196 unique sequences to 114 KEGG pathways and 592 enzymes; gill tissue matched 1,397 unique sequences to 114 KEGG pathways and 674 enzymes; brain tissue matched 1,272 unique sequences to 111 KEG pathways and 620 enzymes (Additional file 7: Table S6).

### Digital gene expression analysis

The cellular response to temperature stress in fish is highly conserved, and the unique evolutionary history and resulting physiological response to elevated temperature previously reported in *T. bernacchii* provides a useful backdrop to discuss the findings from our study. It should be noted, that this study was not designed to explicitly test the effects of temperature on gene expression, since RNA from individuals within a thermal acclimation treatment was pooled prior to library construction. However, an initial qualitative comparison can be performed, and the results compared to previous studies that have performed more quantitative gene expression analyses. To simplify the expression analysis for comparison of tissue specific changes in gene expression across treatments, we limited our consideration to only those unigenes annotated with either a BLAST result, functional mapping, or full GO annotation. We then characterized the relative changes in tissue specific expression patterns in −1.5°C (control) and +4°C acclimated fish. After screening to remove data pairs that did not exhibit expression in either treatment, we achieved our final expression datasets for each tissue. In all, the expression levels of 6,656 unigene pairs were compared in the liver in which we found 1,685 unigenes were down-regulated 2-fold or greater while 3,862 were found to be up-regulated 2-fold or greater (Additional file 8: Table S7). For gill tissue, comparison of the expression levels of 10,084 unigene pairs showed 4,369 unigenes were down-regulated and 3,065 unigenes were up-regulated at least 2-fold or greater (Additional file 8: Table S7). For expression analysis of the brain tissue we compared the expression levels of 9,044 unigene pairs, of which 3,347 were down-regulated and 3,965 were up-regulated at least 2-fold (Additional file 8: Table S7).

Below we discuss the general gene expression trends that emerged from our preliminary RNAseq analyses with respect to the most highly expressed gene families under ambient conditions. In addition, in order to more fully interpret the effects of long-term acclimation to elevated temperatures on *T. bernacchii*, we extended our analysis beyond that of the most highly represented gene families, and in doing so, focused on three particular areas that encompass the cellular response to elevated temperature observed in *T. bernacchii*.

### Tissue specific gene expression profiles under ambient conditions

Prior studies characterizing the gene expression profiles of nothothenioid fishes held under ambient conditions found tissue specific transcriptomes that were dominated by a few highly expressed transcripts [23]. We found a similar pattern among our tissues sampled from *T. bernacchii*, albeit to a lesser degree with the 10 most highly expressed annotated unigenes in the liver, gill and brain tissues representing ~8.2%, ~3.2% and ~2.67% of total transcripts respectively (Additional file 8: Table S7). Similar to results reported for the liver transcriptome of *D. mawsoni*, we found apolipoproteins and fibrogen chains were among the most highly expressed genes in the liver of *T. bernacchii* under ambient conditions (14 kda apolipoprotein = 1.19%, apolipoprotein A-I = 0.66%, apolipoprotein b-like protein = 0.57%, and fibrogen α chain-like = 1.70% of total transcripts) [23]. This over-representation appears to be further exaggerated by long-term acclimation to elevated temperatures. Analysis of the liver transcriptome of fish acclimated to +4°C for 28 days showed apolipoproteins were considerably up-regulated with the 14 kda apoliprotein undergoing a 7.1-fold increase to represent 5.10% of all transcripts and apolipoprotein A-I undergoing a 11.5-fold increase to represent 4.65% of all transcripts (Additional file 8: Table S7). Given their role in lipid mobilization and transport, it seems plausible this significant increase in apolopiproteins is playing an important role in offsetting the rapid increase in metabolic demands previously associated with the acclimatory response of *T. bernacchii* to elevated temperature [30].

In addition, we found similar patterns of gene expression in the brain tissue of *T. bernacchii* as those found in *D. mawsoni.* Chen and colleagues report a number of transcripts that are involved in the protection, maintenance and repair of neural tissue, including S100β and ependymin-1, which are highly represented in the transcriptome of *D. mawsoni* brain tissue [23]. Under ambient temperature conditions, S100β and ependymin-1 comprise 0.30% and 0.18% respectively of the expressed genes in *T. bernacchii* brain tissue, and when exposed to a thermal stress, the expression of these two transcripts increased significantly. In fish acclimated to +4°C, S100β increase over 13-fold, representing 2.21% of all expressed transcripts and ependymin-1 increased by greater than 7-fold to represent ~2% of all expressed transcripts in the brain (Additional file 8: Table S7). Although only semi-quantitative at this point, these data suggest *T. bernacchii* maintain a strong capacity to respond to environmental conditions perturbing to neural function.

The gill tissue displayed a somewhat different trend with respect to the highly expressed transcripts found in the ambient and +4°C acclimated fish. In the gill tissue from fish acclimated to +4°C, we saw an overall down-regulation of many of the most highly expressed transcripts found in ambient fish including, 60s ribosomal proteins (8.8-fold), zinc finger proteins (14.3-fold), MAPKs (4.7-fold) and translation initiation factor eif2b (3.4-fold) (Additional file 8: Table S7). Interestingly, many of these genes were reported to show a moderate up-regulation (<1.5-fold) after an acute heat shock event (4 h at +4°C) in a previous study employing a transcriptome wide analysis of *T. bernacchii*[31]. The differences in gene expression profiles between these two studies likely capture the transition that occurs between immediate response to stress and the long-term physiological adjustments that follow. The general down-regulation of gene families involved in transcription and translation in our long-term treatment may indicate a diminished capacity for protein turnover at elevated temperatures. However, despite a general down-regulation of one of the cells major energy consuming pathways in the gill tissue, we see a 2.5-fold up-regulation of cytochrome c oxidase I and a 1.5-fold up-regulation of cytochrome b (Additional file 8: Table S7) which mirrors the increased capacity for aerobic metabolism found under conditions of acute thermal stress in *T. bernacchii*[31]. These findings, along with the strong up-regulation of apolipoproteins in the liver, suggest that mobilization of energy stores and ATP production continue to play a central role in the capacity of *T. bernacchii* to mitigate the effects of elevated temperatures long-after cellular restructuring has likely occurred.

### Protein homeostasis and the heat shock response (HSR)

**Figure 5:**
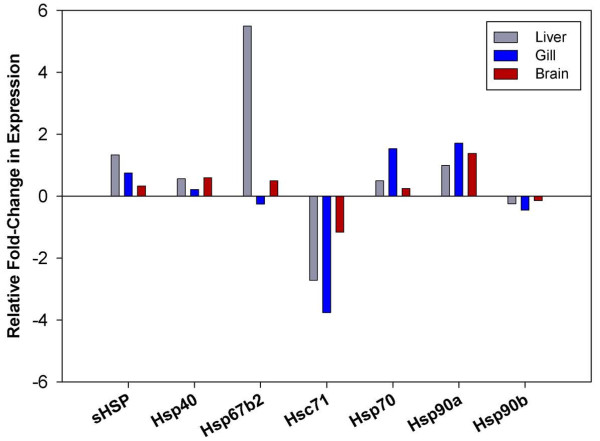
**Differential expression of Heat Shock Protein families in *T. bernacchii.*** Relative fold-change in expression of annotated HSPs in +4°C acclimated fish compared to control fish were obtained from the RNAseq analysis and directly compared across liver (grey), gill (blue) and brain (red). HSPs were grouped by gene family and the relative fold-change computed by averaging the change in expression of gene family constituents.

Heat shock proteins (HSPs) have long been known to play a significant role in folding of nascent polypeptides as well as the rescue and refolding of proteins under conditions of cellular stress [42,43]. Despite the functional roles for these protein families being highly conserved across all taxa, Antarctic notothenioids display divergent expression patterns that might be related to environmental constraints. Previous studies have observed that inducible HSP isoforms and protein-specific chaperones are continuously up-regulated in several notothenioid species, potentially indicating a persistent system-wide requirement for mitigating the denaturing effect of the constant cold of the Southern Ocean [11,23,44]. As a result of this persistent elevation of the heat shock response (HSR), it is believed that the extremely cold adapted notothenioids, including *T. bernacchii,* are no longer capable of further up-regulating inducible isoforms of HSPs when exposed to thermal stress [10,11]. To see if similar trends were identified in our long-term acclimations of *T. bernacchii*, we compared the level of expression for several molecular chaperones involved in the HSR. Two particular HSPs previously found to be up-regulated in fish exposed to chronic high temperature are Hsp70 and Hsp90 [45]. Consistent with previous findings for acute thermal stresses, we found that Hsp70 expression was generally insensitive to long-term acclimation at elevated temperature with only moderate changes detected (≤ 1.5-fold in all tissues, Figure 5). Similarly, the inducible isoform of Hsp90 (Hsp90α) also showed only small variations in expression in all three tissues (liver 1-fold, gill 1.7-fold, brain 1.4-fold) (Figure 5). Also seen with the Hsp70 molecular chaperone, gill tissue appeared to be the most responsive, which may be attributable to the direct contact of these cells to the water and thus environmental variations. Overall, our findings for the expression patterns of HSPs in *T. bernacchii* reflect previous findings and further substantiate the belief that much of its ability to mount a HSR has been lost [11,31,44]. Intriguingly, although no significant changes were seen in the inducible HSP isoforms, we did observe a significant decrease in expression levels of the constitutive isoform, Hsc71, in liver and gill (2.7 & 3.7-fold respectively). This down-regulation of Hsc71 was also accompanied by moderate decreases in the constitutively expressed Hsp90β (Figure 5).

In addition to the major molecular chaperone families represented in the transcriptome, we also identified a number of unigenes with homology to several members of the α-crystallin-type super family, collectively referred to as small heat shock proteins (sHSPs). Although sHSPs are known have similar cytoprotective effects as those found with larger size class molecular chaperones, they are often found expressed in lower abundances and in some instances their function may be non-essential for cell survival [46-48]. In our study, we found the sHPSs showed tissue specific responses with sHSPs in the liver displaying the greatest magnitude change. After acclimation to +4°C, transcript levels for Hsp27/B1, Hsp30/B11 and α-crystallin all showed a 2-fold or greater up-regulation in liver tissue. However, in gill and brain tissue expression of these sHSPs were either not detected or down-regulated, except in the case of α-crystallin in the brain tissue which displayed a 3-fold increase (Additional file 8: Table S7).

As this is only a preliminary characterization of the transcriptome of *T. bernacchii* under chronic thermal stress, we are unable to elucidate the importance of the trends identified with respect to the expression of HSPs. However, in combination with the expression patterns of the transcriptional machinery mentioned above, we speculate this could be related to an overall decrease in transcription with long-term acclimation to elevated temperatures, potentially as a means to conserve energy. We have previously shown metabolic rates in *T. bernacchii* are initially elevated when acclimated to +4°C but gradually return to baseline levels [30]. A general reduction in transcription rates could represent physiological trade-offs associated with long-term physiological adjustments necessary to reduce the cellular demand for oxygen. Alternatively, these reductions in capacity for protein synthesis and chaperoning may represent hallmarks of temperature compensation at the cellular level in these fish. These results represent potentially novel insight into the thermal acclimation of these fish and highlight the sensitivity and utility of these approaches for understanding physiological responses in this system.

### Cellular stress response (CSR)

The cellular stress response (CSR) is a conserved defense reaction activated at the cellular level when exterior forces cause strain on an organism [49]. The heat shock proteins that serve as molecular chaperones and the HSR described above are probably the most well-known constituents of the CSR, but there are a number of additional cellular pathways involved in the cell’s response to stress that are likely co-regulated, including: redox regulation proteins, DNA damage sensing/repair proteins, protein degradation proteins, fatty acid/lipid metabolism proteins and energy metabolism proteins [see 49]. Notothenioid fishes are known to significantly elevate their resting metabolic rates at these temperatures, which could in turn lead to a significant increase in the potential for oxidative damage to macro molecules [30]. Thus, in organisms adapted to extreme environments such as the notothenioids where the HSR has largely been lost, these additional CSR mechanisms could play a more significant role in maintaining cellular homeostasis.

**Figure 6:**
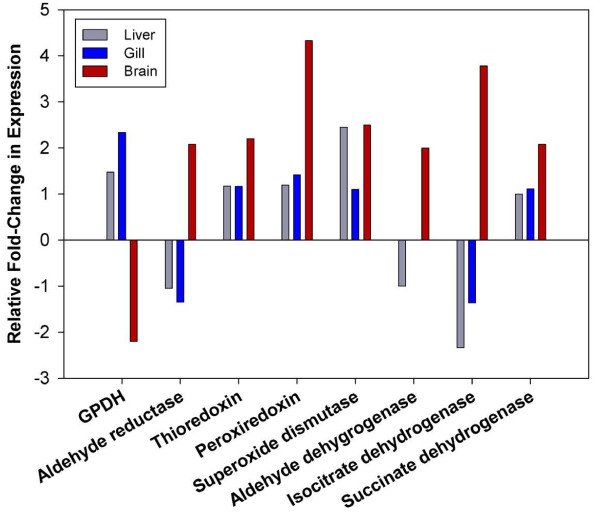
**Differential expression of Redox Regulator Proteins in *T. bernacchii.*** Relative fold-change in expression of annotated redox regulator proteins in +4°C acclimated fish compared to control fish were obtained from the RNAseq analysis and directly compared across liver (grey), gill (blue) and brain (red). Redox proteins were grouped by gene family and the relative fold-change computed by averaging the change in expression of gene family constituents.

The alteration of cellular redox potential due to stress is a major trigger of the CSR and the expression of certain reductases, redoxins and dehydrogenases signify a robust CSR to changing conditions [49,50]. We analyzed these redox regulators using our dataset to provide preliminary insight into the broader CSR of *T. bernacchii*. Each tissue demonstrated a general trend of up-regulation of redox regulators with all but three of the annotated redox regulators being up-regulated in at least two tissues (aldehyde reductase, aldehyde dehydrogenase, isocitrate dehydrogenase), and 4 of 8 up-regulated in all three tissues (thioredoxin, peroxiredoxin, superoxide dismutase, succinate dehydrogenase) (Figure 6). The brain tissue displayed the greatest magnitude of response for redox regulators, with 7 of 8 redox regulators up-regulated 2-fold or greater compared to the liver in which only 5 were found up-regulated and only 1 redox regulator up-regulated 2-fold or more (Figure 6). While *T. bernacchii* does appear to up-regulate redox regulators as part of its CSR, much like the HSPs, the ability to increase the expression of redox regulators may be moderated to some extent and is highly tissue specific.

### High mobility group B1 protein and its role as a global temperature sensor

Previous studies have indicated that the high mobility group b1 (HMGB1) protein, which has a key function in the assembly of transcription initiation and enhanceosome complexes, may serve as a gene expression temperature sensor in fish [45]. When subjecting the killifish (*Austrofundulus limneaus*) to heat shock conditions, the killifish responded by reducing its expression of HMGB1 [45]. The expression levels of HMGB1 in *T. bernacchii* support the idea that HMGB1 is highly responsive to temperature and may serve as a gene expression temperature sensor in fish. However, HMGB1 expression patterns in *T. bernacchii* dramatically differ with those reported for *A. limneaus*. We found HMGB1 undergoes substantial up-regulation when exposed to chronic thermal stress, not down-regulation as seen in the killifish. In all three tissues characterized in this study, HMGB1 showed a 2-fold or greater increase in expressed transcripts when acclimated to elevated temperatures. If we consider the functional role of HMGB1 we might be able to draft some potential insight into these contradictory results.

HMGB1 has two functional roles, an intra-nuclear function where it positively regulates transcription by affecting chromatin structure [51], and an intracellular role (demonstrated in human models) where it can stimulate the inflammatory process by stimulating cytokines from endothelial cells, monocytes and macrophages [52-54]. *T. bernacchii* may be increasing intracellular levels of HMGB1 during exposure to elevated temperatures to increase transcription rates, restructure membrane components and increase aerobic capacity in an effort to meet an elevated demand for oxygen associated with acclimation to these temperatures [30]. Alternatively, it is possible that the increased expression of HMGB1 is a result of inflammation due to cellular damage sustained from the +4°C acclimation temperature. While it is unclear if the differences in HMGB1 regulation in *T. bernacchii* and *A. limneaus* are representative of the adaptive differences between the species, or potentially related to the difference in acclimation times used within the two studies, these data further supports the case for HMGB1 as an important regulator of thermal stress response in fish.

## Conclusion

To our knowledge this is the first large-scale effort aimed at sequencing and assembling the transcriptome of a species from the ecologically important subfamily Trematominae. With this dataset, we now have an annotated transcriptome from at least one member of all three major subfamilies in Notheniidae. Our sequencing, assembly, and annotation efforts yielded an annotated transcriptome library for *T. bernacchii* containing 30,107 unigenes of which 13,003 possess strong homology to known proteins. The subsequent application of this library to characterize tissue specific changes in relative abundance using raw sequencing reads allowed us to observe the transcriptomic composition of a polar organism in much greater detail than previously available. Our preliminary RNAseq analysis of a portion of the CSR demonstrates the potential of digital gene expression analysis to greatly increase our understanding of notothenioids, and polar organisms in general, on a genomic level.

Continued efforts to refine these resources for *T. bernacchii* may well serve to establish these unique fish as a ‘model’ organism for research in polar adapted species and as an important part of a greater effort to increase the genomic resources for polar organisms that inhabit the extreme, yet static polar environments like the Southern Ocean. As global climate change accelerates, and its effects are experienced most rapidly and dramatically at high latitudes [55], continued generation of these resources will prove vital to our understanding of the adaptive potential of endemic polar organisms.

## Methods

### Tissue collection and RNA extraction

All procedures were conducted in accordance with the Animal Welfare Act and were approved by the University of South Carolina Institutional Animal Care and Use Committee (ACUP protocol # 100377). To obtain a broad representation of genes expressed in various tissues under normal and stressed conditions, we used gill, liver and brain tissue that had been collected from a total of ten different fish acclimated to either −1.5°C or +4°C (n = 5 fish per treatment) for 28 d in flow-through seawater tanks located in the Crary Science and Engineering Center at McMurdo Station Antarctica. Immediately after euthanizing the fish, tissues were excised in a −2°C environmental chamber and flash frozen in liquid nitrogen and shipped back to our home institution on dry ice where they were stored at −80°C until used. Total RNA from approximately 100 mg of frozen tissue was extracted using TRIzol (Invitrogen) following the manufacturer’s recommendations. The RNA was further cleaned by re-suspending in 0.1 ml of RNase/ DNase-free water and adding 0.3 ml of 6 M guanidine HCl and 0.2 ml of 100% ethylalcohol (EtOH). The entire volume was loaded onto a spin column (Ambion) and centrifuged for 1 min at 12,000 × g at 4°C. Flow-through was discarded, and filters were washed twice with 0.2 ml 80% EtOH. RNA was eluted off of the filters twice with 0.1 ml of DEPC-treated water. RNA was precipitated by the addition of 0.1 vol of 3 M sodium acetate (pH 5.0) and 2.5 vol of 100% EtOH, mixed by inversion of tubes and placed at −80°C for 1 h. After this period, tubes were centrifuged at 12,000 × g for 20 min at 4°C. Pellets were washed twice with 80% EtOH and re-suspended in 30 μl of RNase/ DNase-free water. Lastly, RNA was DNase treated at 30°C for 10 min. After quality assessment and determination of specific concentration were conducted using an Agilent 2100 BioAnalyzer and pico-green assay respectively.

### cDNA library preparation and 454 sequencing

For each tissue, 10 μg of total RNA from all individuals within a thermal acclimation treatment (n = 5 fish) was pooled and tissue specific, double–stranded cDNA libraries were synthesized using the SMART cDNA synthesis and amplification method [56]. To increase the representation of lowly expressed transcripts, SMART-prepared cDNA was then normalized using duplex-specific nuclease (DSN) method [56,57] that was modified for downstream compatibility with Roche FLX titanium chemistry and 454 pyrosequencing. Briefly, first strand cDNA synthesis was carried out using a SMART cDNA Library Construction kit (Clonetech) according to the manufacturer’s recommended protocol except for the use of a modified CDS-3 M adapter (5’ – AAG CAG TGG TAT CAA CGC AGA GTG GCC GAG GCG GCC TTT GTT TTT TTT TCT TTT TTT TTT VN – 3’). Second strand synthesis and amplification was carried out using an Advantage 2 PCR kit (Clonetech), purified using a QiaQuick PCR purification kit (Qiagen) and quantified with picogreen Quant-iT (Invitrogen). The amplified library was then DSN normalized with a Trimmer-Direct cDNA Normalization kit (Evrogen) followed by amplification of the normalized cDNA. The normalized cDNA was then protinase K treated, *sfiI* digested and size fractionated using chromaspin 400 columns (Clonetech) according to the published SMART cDNA protocol. Lastly, the tissue specific libraries from each thermal acclimation treatment were barcoded with MID-tags and pyrosequencing was carried out using Titanium FLX chemistry on a Roche GS-FLX 454 sequencer by the engencore facility at the University of South Carolina.

### Sequence filtering, *de novo* assembly, and assembly validation

To maximize the relatively high accuracy of Roche GS-FLX 454 sequencing and to ensure a more accurate *de novo* transcriptome assembly we accepted sequences for assembly only after a stringent quality control and trimming process. The 454 sequence primers for the plus strand (CGTATCGCCTCCCTCGCGCCATCAG) and for the minus strand (CTATGCGCCTTGCCAGCCCGCTCAG) were removed. Sequences were then screened for ambiguity and quality; all sequences of low quality (limit = 0.05) or with more than three consecutive ambiguous nucleotides were trimmed or removed if no untrimmed sequence remained.

*De novo* assembly was conducted using the CLC Genomics Workbench *de novo* assembly tool [58]. A word size of 21 and bubble size of 320 were selected as parameters because of the relatively high accuracy of 454 sequencing and the moderate coverage depth (roughly 8X based upon the expectation of 30,000 genes within the transcriptome [59], an average gene length of 1,350 [60], and ~250,000,000 total bases sequenced) to maximize accuracy and unigene length [61]. Reads were mapped back to the unigenes with a minimum percent identity = 0.9 and minimum length = 0.5.

To validate the accuracy of the *de novo* assembly, absent a significantly similar, fully annotated reference genome, the resulting unigenes were gathered into a BLAST database and sequence similarity was compared to all known full and partial protein sequences available for *T. bernacchii* on NCBI unigene database using tBLASTn (*e-*value 10^-10^, word size 3, gap penalty −9, gap extension penalty −2, mismatch penalty −2, match award 1) [62]. The distribution of the *e*-value and greatest percent identity between unigene query and protein result was gathered and greatest percent identity charted to demonstrate the quality and accuracy of the resulting *de novo* transcriptome assembly. A BLAST comparison was also conducted against 9 previously sequenced teleost fish including *Notothenia coriiceps, Chaenocephalus aceratus, Pleuragramma antarcticum, Gasterosteus aculeatus, Takifugu rubripes, Oryzias latipes, Tetraodon nigroviridis, Oreochromis niloticus* and *Danio rerio* to further validate the transcriptome assembly.

### Transcriptome annotation and analysis

The resulting unigenes were loaded in the B2GO software package for further analysis [63]. Using the unigenes as query sequences, BLASTx searches were conducted against the non-redundant protein sequences (nr) database and BLASTn searches were conducted against nucleotide collection (nr/nt) database at NCBI, the acceptance cutoff was an *e*-value of 10^-3^. Unigenes with BLAST results were mapped and annotated using the mapping and annotation functions of B2GO. InterProScan searches were conducted for each unigene [64] and all annotation information was merged into the B2GO interface generating our final gene ontology (GO) annotations. GO annotations were exported from B2GO in Web Gene Ontology (WEGO) format and input into the BGI WEGO Annotation Plotting tool to provide the gene ontology distribution of the *T. bernacchii* transcriptome at GO level 2 [65]. Pathway assignments were determined using the Kyoto Encyclopedia of Genes and Genomes pathway database (KEGG) [41] using a BLASTx threshold cutoff of 10^-5^.

### Tissue specific gene expression

Reads from each tissue (liver, gill and brain) and treatment (−1.5°C control and +4°C acclimated fish) were individually analyzed to characterize the functional representation of expressed genes. The CLC Genomics Workbench RNAseq analysis tool was employed to calculate read expression for each sample using the unigene library as the reference transcriptome. The sequencing reads from each tissue were mapped back to the reference unigenes based upon a minimum similarity of 0.9 and length fraction of 0.5 [66]. Any unigene with at least one successfully mapped sequencing read from either treatment was deemed expressed in that tissue sample. Expressed unigene subsets were created for each tissue type in B2GO to provide tissue specific gene ontology and used to generate a graphical comparison of the transcriptome and tissue specific gene ontology. Furthermore, the enzyme code distribution for each tissue was generated using B2GO and compared between tissues. Lastly, the pathway assignments of the expressed unigenes in each tissue were determined using the KEGG database with a BLASTx *e*-value cutoff of 10^-5^ and compared between the full transcriptome and specific tissues.

### Digital gene expression analysis

The results obtained from the RNAseq analysis of the control and heat shock groups of each tissue were input into the Transcriptomics Experimental Design Tool within the CLC Genomics Workbench to generate a paired samples expression analysis for each tissue. The experimental results were refined by removing any paired sample that did not exhibit expression in at least one treatment. Experimental pairs were annotated using the annotated unigene transcriptome library.

## Competing interests

The authors declare that they have no competing interests.

## Authors’ contribution

SP conceived the study, conducted the temperature acclimation experiments, procured the sequencing data, advised in the assembly, annotation, classification of the data, and the digital gene expression analysis. TH performed the assembly, annotation, classification of the data, and the digital gene expression analysis. Both authors drafted and edited the final transcript.

## Supplementary Material

Additional file 1: Table S1 Percent identity BLAST output. Click here for file

Additional file 2: Table S2 Full transcriptome BLAST2GO data. Click here for file

Additional file 3: Table S3 Full transcriptome WEGO data. Click here for file

Additional file 4: Table S4 Full transcriptome KEGG pathway results. Click here for file

Additional file 5: Figure S1 Full transcriptome enzyme code distribution. Click here for file

Additional file 6: Table S5 Tissue specific BLAST2GO GO data. Click here for file

Additional file 7: Table S6 Tissue specific KEGG pathway results. Click here for file

Additional file 8: Table S7 Digital gene expression analyses data. Click here for file
