# Supplementary figures and images for: De novo assembly and characterization of tissue specific transcriptomes in the emerald notothen, Trematomus bernacchii
Source: BMC Genomics. 2013 Nov 20;14:805. doi: 10.1186/1471-2164-14-805 (PMC3840625; doi:10.1186/1471-2164-14-805)

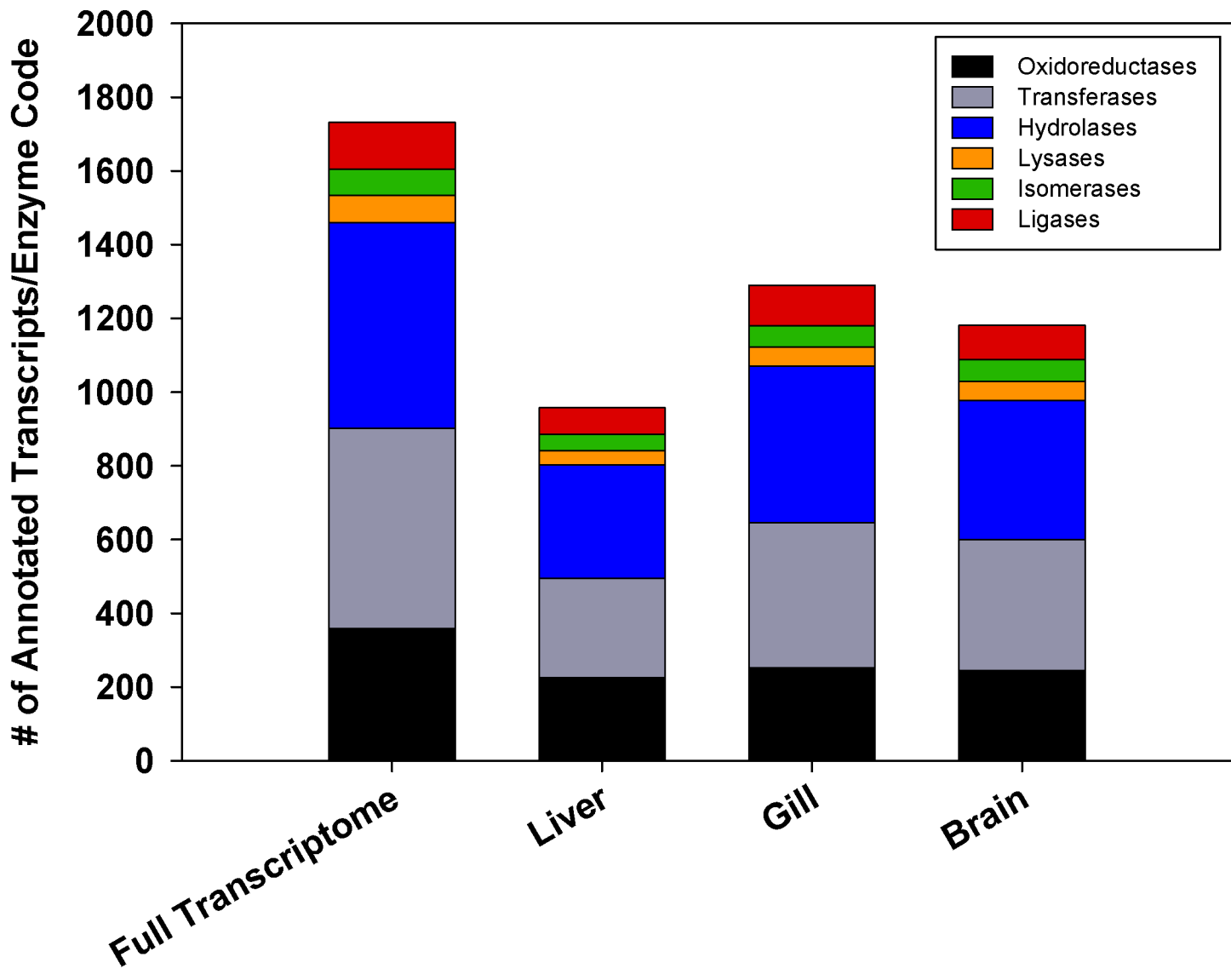

Supplement: Additional file 5: Figure S1 — Full transcriptome enzyme code distribution. [file 1471-2164-14-805-S5.pdf]
